# Quality of life in patients with Graves’ orbitopathy submitted to orbital decompression: comparison between balanced and inferomedial techniques

**DOI:** 10.5935/0004-2749.2023-0296

**Published:** 2024-08-30

**Authors:** Cristiane de Almeida Leite, Thaís de Sousa Pereira, Jeane Chiang, Rodrigo Bernal Moritz, Allan Christian Pieroni Gonçalves, Mário Luiz Ribeiro Monteiro

**Affiliations:** 1 Laboratory of Investigation in Ophthalmology (LIM 33), Division of Ophthalmology Faculdade de Medicina, Universidade de São Paulo, São Paulo, SP, Brazil

**Keywords:** Graves’ ophthalmopathy, Quality of life, Exophthalmos, Strabismus, Diplopia, Decompression, surgical

## Abstract

**Purpose:**

To compare inferomedial wall orbital decompression to balanced medial plus lateral wall orbital decompression in patients with Graves’ orbitopathy in the inactive phase with regard to exophthalmos reduction and the effects on quality of life.

**Methods:**

Forty-two patients with inactive Graves’ orbitopathy were randomly divided into two groups and submitted to one of two orbital decompression techniques: inferomedial wall orbital decompression or medial plus lateral wall orbital decompression. Preoperative and postoperative assessments included Hertel’s exophthalmometry and a validated Graves’ orbitopathy quality of life questionnaire. The results of the two groups were compared.

**Results:**

Compared to preoperative measurement, exophthalmos reduction was statistically significant in both groups (p<0.001) but more so in patients undergoing medial plus lateral wall orbital decompression (p=0.010). Neither orbital decompression techniques increased the visual functioning subscale score on the Graves’ orbitopathy quality of life questionnaire (inferomedial wall orbital decompression p=0.362 and medial plus lateral wall orbital decompression p=0.727), but a statistically significant difference was observed in the score of the appearance subscale in patients submitted to medial plus lateral wall orbital decompression (p=0.006).

**Conclusions:**

Inferomedial wall orbital decompression is a good alternative for patients who do not require large exophthalmos reduction. However, medial plus lateral wall orbital decompression offers greater exophthalmos reduction and greater improvement in appearance (higher Graves’ orbitopathy quality of life questionnaire scores), making it a suitable option for esthetic-functional rehabilitation.

## INTRODUCTION

Graves’ orbitopathy (GO) severely affects patients’ quality of life (QoL) and social functioning and is associated with neuropsychiatric disorders more often than other chronic eye diseases that induce major visual loss^(1)^. GO patients have higher depression and anxiety levels, especially when afflicted by disfiguring exophthalmos, diplopia, and dysthyroid optic neuropathy^(2)^. Limitations in daily activities and negative health perceptions remain for many years after treatment^(3)^.

The first specific QoL questionnaire for GO (GO-QoL)^(4)^ was developed in 1998, making it possible to objectively quantify the loss of QoL in GO patients associated with physical and psychological changes. The GO-QoL is the most extensively validated and widely used questionnaire in this patient population and should be the choice in evaluating primary results in clinical trials^(5,6)^.

The two most commonly employed orbital decompression (OD) techniques are inferomedial wall OD (IM-OD) and balanced medial plus lateral wall OD (ML-OD). Both are considered effective at reducing exophthalmos. However, these techniques have never been compared in a prospective randomized clinical trial with regard to improvement in QoL. In this study, IM-OD and ML-OD were evaluated regarding exophthalmos reduction and postoperative QoL based on GO-QoL scores.

## METHODS

### Study design

A prospective randomized clinical trial was conducted at a single referral outpatient ophthalmology service. The study protocol followed the tenets of the Declaration of Helsinki, and institutional review board approval was obtained. All participants gave their informed written consent.

### Subjects

Forty-two GO patients in the inactive phase and clinical indication for OD were studied. GO was classified according to disease activity based on the Clinical Activity Score (CAS)^(7)^. Patients with CAS <3, clinical stability for at least 6 months, and disease duration >2 years were considered in the inactive phase. Patients exhibited predominantly the myogenic subtype of GO.

Inclusion criteria were (i) GO diagnosis in the inactive phase; (ii) granting of informed written consent; (iii) age ≥21 years; (iv) euthyroidism; (v) exophthalmometry ≥20 mm; (vi) absence of eye abnormalities such as degenerative myopia, microphthalmos, and anophthalmia; (vii) absence of other orbital abnormalities such as previous fractures and congenital defects; (viii) good level of cooperation with study procedures; (ix) ability to comply with the consultation schedule; and (x) absence of contraindications for OD in the preoperative clinical evaluation.

Exclusion criteria were (i) myasthenia gravis; (ii) pregnancy; (iii) previous orbital, strabismus, or eyelid surgery; and (iv) other abnormal eye conditions or symptoms preventing the patient from participating in the study, as per the investigator’s clinical judgment.

### Randomization

Patients were randomly assigned to one of two groups according to the surgical technique (IM-OD or ML-OD). To do so, the first patient examined was asked to draw a lot, initiating the sequence of patients alternating between the techniques. The researcher scheduling the surgery differed from the orbit surgeons and the researcher performing preoperative and postoperative evaluations. The latter researcher and the patient were blinded to the OD technique.

### Surgical techniques

Invariably, IM-OD was performed by one of the authors, whereas ML-OD was performed by another author. Both orbit surgeons had extensive experience in their respective techniques. Both techniques were performed under general anesthesia.

IM-OD was slightly modified in relation to previous descriptions^(8,9,10)^. In short, the medial wall of the orbit was accessed with the transcaruncular approach^(11)^. A C-shaped incision was made vertically just behind the caruncle in the medial conjunctiva, with dissection posteriorly through the subconjunctival tissue and medially in the preseptal plane to the posterior lacrimal crest. The medial wall (the lamina papyracea of the ethmoid bone) was completely dissected and fractured, respecting its superior limit with the frontal bone and posterior limit with the lesser wing of the sphenoid bone. The inferior limit (the junction with the maxillary bone comprising the inferomedial orbital strut)^(8)^ was preserved in its anterior portion. The orbital floor (maxillary sinus roof) was accessed through a fornix transconjunctival incision^(9,10)^ without lateral canthotomy whenever possible. The maxillary fracture was limited to the medial portion relative to the infraorbital groove. The periorbita opening was carefully planned to avoid the recti muscles paths.

The ML-OD technique involved medial and lateral wall decompression while sparing the orbital floor. As in IM-OD, the transcaruncular approach was used to access the medial wall. Access to the lateral wall was achieved as described in the literature^(12,13)^. The superolateral orbital rim was exposed by a lateral incision of the upper eyelid. The lateral wall was dissected, and all three areas of thick bone were sculpted and thinned with a high-speed diamond drill: the lacrimal fossa (to improve visualization), the greater wing of the sphenoid, and, inferolaterally, the zygomatic bone and part of the maxilla. Periorbita incisions enabled orbital fat to herniate into the newly created space.

### Pre-Preoperative and postoperative evaluation

Before surgery and 6 months postoperatively, patients underwent a complete ophthalmologic examination, including Hertel’s exophthalmometry and a self-rated GO-QoL. The presence or absence of binocular diplopia in the nine positions of gaze was scored from 0 to 100 using the Diplopia Questionnaire developed by Holmes et al.^(14)^.

Go-QoL has been validated in 10 languages and is widely used in research^(4,6,15)^. The questionnaire contained 16 questions: 8 referring to the consequences of diplopia and low visual acuity (“visual functioning”) and 8 referring to the psychosocial consequences of changes in physical appearance (“appearance”). The questions were answered on a three-point scale (1=“yes, seriously limited”; 2=“yes, a little limited”; 3=“no, not limited at all”). In this manner, two subscales were obtained, varying from 8 to 24 points. The subscales were transformed with the formula: total score = [(raw score–8)/16] ×100. As a result, each subscale had a score between 0 and 100, with higher scores indicating better QoL.

### Statistical analysis

Statistical analysis was performed using Stata version 15 (StataCorp, College Station, TX, USA) and Statistica version 13 (TIBCO Software, Inc., Palo Alto, CA, USA).

The sample size was established using “exophthalmos reduction” as the main variable. Because the mean ± standard deviation (SD) in the literature was 2.1 mm and the desired effect size, based on clinical judgment, was 1.5 mm, a minimum sample size of 24 eyes in each group was established.

The χ^2^ association test was used to verify equivalent distributions of demographic and clinical variables. Repeated-measures analysis of variance (ANOVA) was used to calculate differences between the two groups regarding mean preoperative and postoperative quantitative parameters. Multiple comparisons, when appropriate, were performed with Tukey’s honestly significant difference (HSD) test. In all analyses, differences were considered statistically significant when p<0.05 (α error=5%).

## RESULTS

### Demographic and clinical variables

Table 1 shows the demographic data and clinical characteristics of the 42 patients included in the study. No statistically significant difference was observed between the groups regarding sex distribution, age, antithyroid stimulating hormone (TSH) receptor antibody (TRAb) dosage, smoking, family history of thyroid disease, treatment for Graves’ disease (GD), or GO treatment.

**Table 1:** Demographic and clinical variables of GO patients submitted to OD

Variable	Category	Group (%)		p-value*
		IM-OD (n=21)	ML-OD (n=21)	
Sex				
Female		15 (71.4)	16 (76.2)	0.726
Male		6 (28.6)	5 (23.8)	
Age ± SD (years)		47.5 ± 12.7	49.9 ± 10.9	0.616
TRAb	Positive	7 (43.8)	8 (47.1)	0.849
Smoking	Yes	9 (42.9)	12 (57.1)	0.355
Family history	Yes	11 (52.4)	8 (38.1)	0.352
**GD treatment**				
Anti-thyroid drugs	Yes	17 (81.0)	18 (85.7)	1.000
Radioiodine therapy	Yes	11 (52.4)	14 (66.7)	0.346
Thyroidectomy	Yes	2 (9.5)	7 (33.3)	0.130
Hormonal reposition	Yes	2 (9.5)	0 (0.0)	0.488
**GO treatment**				
Lubricant eye drops	Yes	14 (66.7)	17 (80.9)	0.292
Corticosteroids	Yes	20 (95.2)	20 (95.2)	1.000
Radiotherapy	Yes	1 (4.8)	1 (4.8)	1.000

* χ^2^ association test.

No major surgical complications (e.g., visual loss, permanent infraorbital dysesthesia, and hypoglobus) occurred in either group.

### Exophthalmos

Twenty-one patients (42 orbits) were submitted to IM-OD and 21 (42 orbits) to ML-OD. The mean preoperative exophthalmometry findings were similar for the two groups (p=0.899). Postoperative reduction on exophthalmometry was significant in both groups (p<0.001) but significantly greater for ML-OD than for IM-OD (3.8 ± 3.1 mm vs. 2.4 ± 1.9 mm; p=0.010; Table 2).

**Table 2:** Hertel exophthalmometry findings in the two study groups (ML-OD and IM-OD) before and after OD

Group	n	Exophthalmometry (mm), mean ± SD (range)		Exophthalmos reduction (mm) (Mean ±), mean ± SD (range)	p-value
		Preoperative	Postoperative		
IM-OD	42	23.9 ± 2.8 (20-30)	21.4 ± 2.9 (14-28)	2.4 ± 1.9 (0.5-8)	<0.001*
ML-OD	42	23.5 ± 2.6 (20-34)	19.6 ± 2.2 (14-24)	3.8 ± 3.1 (0.5-12)	<0.001*
		p=0.899*	p=0.010*		

* Repeated-measures ANOVA/Tukey’s HSD test.

### Diplopia questionnaire

Concerning the Diplopia Questionnaire, the preoperative and postoperative scores for IM-OD were 18.3 ± 25.9 and 25.7 ± 39.3 points, respectively. Similarly, the preoperative and postoperative scores for ML-OD were 16.4 ± 21.1 and 23.0 ± 30.3 points, respectively. These scores were statistically similar preoperatively and postoperatively (p=0.094) and between the two groups (p=0.783)^(16)^.

### GO-QoL

Table 3 shows the two subscales of GO-QoL scores. In the visual functioning subscale, no statistically significant difference was observed between the mean preoperative and postoperative scores or between the two OD techniques (IM-OD p=0.362 and ML-OD p=0.727). In the appearance subscale, there was a statistically significant difference between the mean preoperative and postoperative scores, but only for ML-OD, with an average increase of 20.4 points in the postoperative period (IM-OD p=0.675 and ML-OD p=0.006).

**Table 3:** Comparison of GO-QoL scores preoperatively and after 6 months of OD in 42 GO patients

GO-QoL subscales Group	GO-QoL scores (0–100), mean (SD)		p-value*
	Preoperative	Postoperative	
**Visual functioning**			
IM-OD	82.2 (17.2)	90.5 (13.6)	0.362
ML-OD	87.9 (14.4)	82.8 (19.9)	0.727
**Appearance**			
IM-OD	67.4 (17.6)	73.5 (17.1)	0.675
ML-OD	60.9 (15.7)	81.3 (12.9)	**0.006**

* Repeated-measures ANOVA/Tukey’s HSD test.

## DISCUSSION

The concept of QoL was introduced in 1964. In 1977, QoL was mentioned for the first time as a keyword in the Medline database. QoL is considered an important clinical measure when describing the disease severity^(6)^.

QoL comprises a complex set of physical, mental, and social aspects and must be self-assessed by the patient. Discrepancies between objective clinical measures and the patient’s subjective experience can be explained by the fact that the perception of health and the ability to perform daily activities are not determined by the severity of physical signs and symptoms alone but also by the characteristics of the individual and the environment (including experiences, expectations, awareness, beliefs, motivation, social support, and the doctor-patient relationship)^(6)^.

Moreover, psychological disorders in GO patients may be related not only to changes in physical appearance and visual functioning but also to the detrimental impact of circulating thyroid hormones on the central nervous system. Studies in healthy human brains have revealed that TSH receptors are significantly expressed in cortical and limbic tissues (amygdala, cingulate gyrus, frontal cortex, hippocampus, hypothalamus, and thalamus), suggesting an interlocution between the endocrine and neuropsychiatric systems^(17)^. In GD patients with or without GO, antibodies binding to TSH receptors in the brain can contribute to the development of neuropsychiatric disorders, such as cognitive and emotional impairment^(18)^. GO patients suffer from more mood disorders, depression, and anxiety than patients affected by other chronic diseases and facial disfigurements, especially when burdened by exophthalmos, diplopia, and dysthyroid optic neuropathy^(2)^.

The first systematic assessment of QoL in GO patients was made in 1997 using a questionnaire applicable to various diseases, patients, and populations (MOS SF-24)^(19)^. GO patients scored worse than individuals with diabetes, emphysema, or heart failure and about the same as individuals with inflammatory bowel disease. Interestingly, the score was unrelated to GO duration, activity, or severity.

The long-term effects of GO on QoL were studied with another generic questionnaire (MOS SF-36), which reported that GO patients still reported limitations in daily activities and worse perception of health compared to the normal population even after 11.7 years of treatment; 32% of the patients reported that “their eyes did not look normal”, and 28% were not satisfied with their appearance. These findings suggested that GO should be considered a chronic disease^(3)^.

In 1998, the first GO-QoL was developed^(4)^. The GO-QoL measures the specific aspects of the QoL of GO patients and provides additional information on physiological or traditional biological health conditions^(4)^. As with earlier generic questionnaires, GO-QoL scores were initially only moderately correlated with the clinical parameters of GO activity and severity^(3^, ^4,19)^. The low level of consistency was justified by the subjective nature of the QoL construct. In contrast, more recent studies using the GO-QoL reported greater internal consistency: in patients with long-term GO, the scores of visual functioning and appearance subscales were negatively associated with activity criteria (CAS) and GO severity. Diplopia was reflected in the visual functioning subscale, whereas exophthalmos and asymmetry significantly impacted the appearance subscale^(15,20,21)^.

Subsequent publications have validated the GO-QoL, which is a reliable and reproducible tool with intraclass correlation coefficients >0.80^(22)^.

The effects of GO treatment on GO-QoL scores were assessed by Terwee et al.^(23)^, who reported an increase from 10 to 20 points after radiotherapy or OD and from 3 to 10 points after strabismus and eyelid surgery. An average increase of 6 points in one or both GO-QoL subscales reflected an important improvement in daily activities.

Other GO-specific questionnaires have been proposed: an adaptation of the GO-QoL to the Australian setting^(21)^ and a German version awaiting validation^(24)^, the GO-QLS (a longer and more time-consuming instrument from the USA)^(25,26)^, the NEI VFQ-25 (of uncertain validity and reproducibility), and the Canadian instrument TED-QoL, which is more suitable for patient guidance than for clinical trials^(27)^.

The generic questionnaires (MOS SF-24 and SF-36) and the GO-specific questionnaires (GO-QoL, TED-QoL, etc.) show an important deterioration in the QoL of GO patients due to adverse physical and psychological repercussions. This indicates the need for a multidisciplinary approach involving endocrinologists, ophthalmologists, radiologists, psychiatrists, and neurologists and a treatment regime tailored to improve QoL, perception of orbital disease, and adherence to medical recommendations and surgical treatment^(1,2)^.

Despite efforts to define specific criteria of GO activity and severity to improve patient management, greater attention should be paid to psychosocial aspects. The assessment of patients’ QoL should be included in the assessment of GO severity and considered an indicator of treatment success to improve the patient’s clinical outcome and well-being^(1)^. In 2006, the EUGOGO recommended using GO-QoL as a measure to evaluate responses to treatments in clinical trials^(28)^.

In this study, preoperative and postoperative scores of the Diplopia Questionnaire were statistically similar regardless of the OD technique employed. These findings demonstrated that both techniques are deemed safe concerning the risk of new-onset or worsening diplopia^(16)^. However, no significant difference was observed on the visual functioning subscale between preoperative and postoperative periods or between OD techniques. This may be explained mainly by complaints about ocular surface issues and diplopia, which at this stage involved no correction of strabismus or eyelid surgery^(29)^.

In the appearance subscale, there was a statistically significant difference in the mean scores between preoperative and postoperative periods only in the group submitted to ML-OD, with an average increase of 20.4 points. According to Terwee et al.^(23)^, an average increase of 6 points in one or both GO-QoL subscales by patients was perceived as beneficial and associated with considerable improvement in daily activities. However, after invasive treatments such as OD, the score might increase by ≥10 points^(30)^. These findings suggested that ML-OD is efficient at cosmetically correcting disfiguring exophthalmos, yielding positive impacts on psychosocial functions^(24)^.

This study is subject to limitations primarily due to the heterogeneous nature of GO. Because the sample was relatively small, randomization may not adequately match both groups. Despite the proficiency of both surgeons in their respective techniques, variations in their expertise may have introduced a bias element that potentially influenced the study outcomes. Another limitation pertained to the study’s exclusive execution within a tertiary hospital setting, raising the possibility that the sample might not accurately represent more typical GO cases. Finally, it is important to emphasize that the choice of a specific OD technique should not only consider GO-QoL scores but also consider the individual clinical and radiological characteristics of each patient and the surgeon’s expertise.

To obtain the best therapeutic results in GO, the approach must be focused not only on the clinical aspects of the disease but also on the impact of GO on patients’ QoL and their psychosocial well-being^(5)^. To achieve this, a multidisciplinary approach is necessary^(1)^. GO has a marked negative effect on the QoL, even many years after treatment. These findings suggested that GO should be considered a chronic disease; psychological monitoring and support should be maintained for these patients even after clinical and surgical treatments^(3)^.

IM-OD and ML-OD achieved similar scores on the visual functioning subscale of the GO-QoL, but ML-OD was associated with better scores on the appearance subscale, suggesting that ML-OD is a good option for the cosmetic functional rehabilitation of patients with disfiguring exophthalmos.
